# Supplementary material for: Heterogeneity of use, access, and retention of insecticide-treated nets: Implications for subnational tailoring to maximise malaria control
Source: eLife. 2026 Aug 20;14:RP108745. doi: 10.7554/eLife.108745 (PMC13493128; doi:10.7554/eLife.108745)
Supplement: Peer review history

# Editor report: eLife Assessment

**DOI:** 10.7554/eLife.108745.4.sa0

**Authors:** Marcelo U Ferreira

**Affiliations:** University of São Paulo Brazil

**Keywords:** Compelling, Fundamental

This work presents **fundamental** findings on the probability of use and access of inseticide-treated nets and evaluates the effectiveness of different distribution strategies in six African countries. The authors propose a sophisticated methodological framework that accounts for many sources of uncertainty, providing **compelling** strength of evidence.

# Referee report: Reviewer #1 (Public review)

**DOI:** 10.7554/eLife.108745.4.sa1

[Editors' note: this version has been assessed by the Reviewing Editor without further input from the original reviewers. The authors have addressed the comments raised in the previous round of review.]

This paper aims to improve the accuracy of predictions of the impact of ITN strategies by developing a method to estimate duration of ITN access and use over time on a subnational scale from cross-sectional survey data and the numbers ITNs received annually. The subnational estimates are then input into a mathematical model to predict clinical cases under different ITN distribution strategies.

Strengths:

The approach is novel and addresses a useful and timely topic. It makes use of available routine data, and has considered all of the relevant components of ITN distributions.

The authors have made revisions, particularly to the methods, appendices and title - leaving the paper easier to follow, and with a clear, consistent aim. The assumptions are clearly stated.

# Referee report: Reviewer #2 (Public review)

**DOI:** 10.7554/eLife.108745.4.sa2

Summary:

The authors design a custom Bayesian model to estimate the probabilities of access, use and use given access of insecticide-treated nets in six African countries, providing sub-national estimates and inferring the average duration of ITN use and access. An individual-based model was employed to simulate malaria epidemics and estimate the effectiveness of different ITN distribution strategies. The study finds that the mean probability of use or access did not reach 80% (a universal coverage formerly targeted by WHO) for any of the regions even for biennial campaigns, demonstrates that switching from triennial to biennial distribution campaigns increases population use by 7.9%, and evaluates the impact of employing more efficient ITNs on *P. falciparum* prevalence.

Strengths:

The authors developed a data-driven model that accounts for data collection imperfections and sources of uncertainty while differentiating between ITN use and access. They developed a methodology to infer the timing of mass campaign from publicly available data instead of assuming fixed dates. The probability of use given access allows determining the regions where ITN distribution is least effective. This work can help better inform future interventions by identifying regions where increasing mass campaign frequency or employing better ITNs are most effective. Finally, in addition to insights on ITN access and use for the six countries analyzed, the paper contributes with a methodological framework that can likely be extended to other countries.

# Author comment: Author response

**DOI:** 10.7554/eLife.108745.4.sa3

**Authors:** Andrew C Glover, Hannah Koenker, El Hadji Amadou Niang, Kate Kolaczinski, Thomas S Churcher

**Affiliations:** Imperial College London London United Kingdom; PMI REACH Malaria, PATH Washington D.C. United States; Université Cheikh Anta Diop Dakar Senegal; Global Fund to Fight AIDS, Tuberculosis and Malaria Geneva Switzerland; Imperial College London London United Kingdom

The following is the authors’ response to the original reviews.

**Public Reviews:**

**Reviewer #1 (Public review):**

This paper aims to improve the accuracy of predictions of the impact of ITN strategies by developing a method to estimate duration of ITN access and use over time on a subnational scale from cross-sectional survey data and the numbers ITNs received annually. The subnational estimates are then input into a mathematical model to predict clinical cases under different ITN distribution strategies.

Strengths:

The approach is novel and addresses a useful and timely topic. It makes use of available routine data, and has considered all of the relevant components of ITN distributions.

The authors have made revisions, particularly to the methods, appendices and title - leaving the paper easier to follow, and with a clear, consistent aim. The assumptions are clearly stated.

Weaknesses:

The weaknesses are shared with other models of a similar complexity - it is not easy for a casual reader to fully understand the model or the implications of the assumptions which were required to be made. That routine data is used is good for availability, but data quality may be an issue in some places.

**Reviewer #2 (Public review):**

Summary:

The authors design a custom Bayesian model to estimate the probabilities of access, use and use given access of insecticide-treated nets in six African countries, providing sub-national estimates and inferring the average duration of ITN use and access. An individual-based model was employed to simulate malaria epidemics and estimate the effectiveness of different ITN distribution strategies. The study finds that the mean probability of use or access did not reach 80% (a universal coverage formerly targeted by WHO) for any of the regions even for biennial campaigns, demonstrates that switching from triennial to biennial distribution campaigns increases population use by 7.9%, and evaluates the impact of employing more efficient ITNs on *P. falciparum* prevalence.

Strengths:

The authors developed a data-driven model that accounts for data collection imperfections and sources of uncertainty while differentiating between ITN use and access. They developed a methodology to infer the timing of mass campaign from publicly available data instead of assuming fixed dates. The probability of use given access allows determining the regions where ITN distribution is least effective. This work can help better inform future interventions by identifying regions where increasing mass campaign frequency or employing better ITNs are most effective. Finally, in addition to insights on ITN access and use for the six countries analyzed, the paper contributes with a methodological framework that can likely be extended to other countries.

Weaknesses:

Since the models employed are rather complex, the methodology description may be hard to follow for some readers. In addition, the models assume many hypotheses, including exponential decay of ITN use/access and narrow prior distributions. It is worth noting that, in the revised version of the manuscript, the authors justified the choice of exponential decay and narrow prior distributions, and made a significant effort to clarify the methodology and the model equations.

Comments on revised version:

I appreciate the improvements made to the text. The methodology description is much clearer now. I have no further suggestions.

We thank the reviewers and editors for their constructive and insightful comments throughout the review process.

**Recommendations for the authors:**

**Reviewer #1 (Recommendations for the authors):**

P8 'Improving ITN use' L218

The numbers do not seem add up to me. "...increases across all settings of 14.5% (95% CrI:14.5, 14.6), from 41.7%% to 49.6%. Greater increases are predicted to be seen for ITN use with mean use across all settings increasing from 58.0% to 66.2%, an increase of 19.5% CrI (95% CrI:19.5, 19.6)."

Thank you for highlighting this. We have reviewed all reported results on mean use, access and use given access. The previous text reported a mixture of absolute and relative % changes, as well as a mixture of raw mean estimates across all regions and population-weighted means across regions. In the extract above we had inadvertently mixed different metrics. Given administrative-one regions can vary notably in population between different countries, we have ensured estimates are now consistently reported as population-weighted means, so that countries with finer-scaled administrative-one regions, such as Burkina Faso, do not artificially bias a raw mean estimate across all sub-national regions. We have also reported % changes as absolute percentage-point increases throughout, rather than relative ones to improve clarity.

Methods p18: There is notation in the text which does not seem to be explained. It is in the appendices, but the appendices should be optional extra information rather than essential for understanding.

We have reviewed the text in the main methods to check notation explanations. Following this, we have removed a use of subscript $i$, which is only used in the appendices to explicitly indicate region-specific parameters, and have clarified that lambda is a decay parameter.

There are assumptions made and these are clearly explained in the text. However, how much the highlighted results rest on the assumptions was not clear, and there was little on this in the discussion.

For example, it might seem disappointing that changing from triennial to biennial ITN campaigns would only lead to an increase from 41.7% to 49.6%. The most important assumptions driving this could be clearer. Additionally, after reading I was not sure what the likely consequences of the assumption that ITN are used continuously were.

We have added some additional text “to the discussion to clarify the modest predicted increase under biennial campaigns may, in part, be influenced by our assumed exponential loss function, and have highlighted that larger increases in mean use could plausibly be predicted under alternative ITN loss functions”. However, we have also commented that our mean use estimates are broadly in agreement with time series modelled estimates by Bertozzi-Villa et al. (2021) who utilised a sigmoidal/smooth-compact loss function.

In relation to the assumption of continuous use, we have added additional text in the ‘Historical use, access and retention times’ methods section to clarify that “if ITN use were systematically higher during high-transmission rainy seasons, our assumption of continuous use may underestimate the protective impact of ITNs during these periods”. As stated at the start of that paragraph, the data available from DHS surveys was too infrequent to investigate seasonal fluctuations.

P14 The text seems to imply that current transmission intensity is the only criterion for decisions about interventions. However, it is likely that the reasons for the current intensity, such as vectorial capacity, historical transmission and interventions should also play a role. The wording could reflect this.

We have added additional text to clarify that current transmission intensity should not be treated as the only criterion for deprioritisation decisions:

“However, current incidence should be considered alongside the factors that gave rise to that transmission intensity, with caution exercised when deprioritising mass campaigns in areas where historically higher transmission may currently be suppressed by high ITN access, high use given access, or other interventions.”

Minor points

There are several definite numbers in the first paragraph of the Introduction - these are estimates rather than the absolute truth, but the wording does not acknowledge that there is uncertainty.

We have made minor edits to clarify that these values are estimates rather than exact quantities. Measures of uncertainty, such as credible intervals were not always possible to source; for example, some of these are median estimates inferred from figures in Bertozzi-Villa et al. (2021).

L634 typo - logisitic

Now corrected.

L1731 typo https://https://

Now corrected.

L881 "access at random" - perhaps not the easiest for non-modelers

We have re-written this to clarify “when ITNs in a household can provide access to more individuals than the number of users, access is assigned at random to non-users within each household under our framework”.

Appendix 1, table 1: Using alpha for both age and also overdispersion on use or access is of course valid, but I found it a little confusing.

To avoid confusion, we have added the following clarification in brackets:

“Meanwhile, the overdispersion parameter, $\alpha_i^0$ (unrelated to the notation for ITN age), controls the variability of the probability of individual access around the mean”

I suspect that the model was actually fitted in Stan via the R interface rstan (L589, L1151 and elsewhere).

We have now clarified this throughout.
